# Anti-*Vibrio parahaemolyticus* Mechanism of Hexanal and Its Inhibitory Effect on Biofilm Formation

**DOI:** 10.3390/foods14040703

**Published:** 2025-02-19

**Authors:** Qiuxia Fan, Mengge Ning, Xuejun Zeng, Xiangxiang He, Zhouya Bai, Shaobin Gu, Yahong Yuan, Tianli Yue

**Affiliations:** 1 College of Food and Bioengineering, Henan University of Science and Technology, Luoyang 471023, China; 9906488@haust.edu.cn (Q.F.); xianghe2009@126.com (X.H.); spbaizhouya@163.com (Z.B.);; 2 College of Food Science and Engineering, Northwest A&F University, Xianyang 712100, China; nmgning@163.com; 3 College of Food Science and Technology, Northwest University, Xi’an 710069, China

**Keywords:** *Vibrio parahaemolyticus*, hexanal, antibacterial mechanism, biofilm formation

## Abstract

*Vibrio parahaemolyticus* (*V. parahaemolyticus*) is one of the most prevalent foodborne pathogens worldwide. Hexanal is a natural aldehyde derived from plants. In this study, the antimicrobial and antibiofilm activities of hexanal against *V. parahaemolyticus* were investigated. Hexanal inhibited *V. parahaemolyticus* growth with a minimum inhibitory concentration (MIC) of 0.4 mg/mL. Hexanal (2 MIC and 4 MIC) increased the leakage of protein and lactic dehydrogenase, reduced intracellular ATP concentration, damaged membrane integrity, and induced abnormal *V. parahaemolyticus* morphology and ultrastructure. The results of colony enumeration suggested that hexanal exhibited bactericidal action against *V. parahaemolyticus* in different culture mediums and food systems (Spanish mackerel meat and shrimp paste). At 1/8 MIC and 1/4 MIC, hexanal inhibited biofilm formation of *V. parahaemolyticus*, as evidenced by crystal violet staining assay and scanning electron microscope (SEM) observation. Moreover, hexanal reduced the levels of extracellular polysaccharide, extracellular protein, and cyclic di-guanosine monophosphate (c-di-GMP) in *V. parahaemolyticus*. The result of real-time quantitative polymerase chain reaction (RT-qPCR) indicated that hexanal downregulated the expression of genes critical to *V. parahaemolyticus* biofilm development. This study provides a promising alternative for *V. parahaemolyticus* control and is conducive to promoting the application of hexanal in the food field.

## 1. Introduction

*V. parahaemolyticus* is a Gram-negative enteric pathogen first found in Japan and has become a primary causative agent of seafood-borne food poisoning outbreaks around the world [1]. *V. parahaemolyticus* is moderately halophilic and grows well in environments with 2.5~3.5% NaCl [2]. Marine products (such as crustaceans, fish, mollusks, and edible marine algae) are the main vehicles for *V. parahaemolyticus* transmission [3,4]. Previous reports demonstrated that some *V. parahaemolyticus* isolates from seafood samples possessed virulent genes [5,6]. For example, a recent survey isolated *V. parahaemolyticus* strains positive for *trh* and *tlh* in sea snail samples collected from a traditional Chinese market in Qingdao City [7]. Therefore, improper consumption of such seafood has a great chance of inducing food poisoning, mainly characterized by diarrhea, abdominal pain, and fever. In addition, *V. parahaemolyticus* also exists in nature in the form of a biofilm, which is a complex microbial community adhering to contact surfaces (such as shellfish, crab, shrimp, stainless steel, and glass, etc.) and is extremely challenging to eliminate [8]. An extracellular matrix outside the structure can provide a protective barrier for *V. parahaemolyticus* cells inside, which grants biofilm a strong resistance to environmental stresses and also makes it a potential inducement of cross-contamination in food processing [9,10]. Consequently, it is of practical significance to seek effective methods to counter *V. parahaemolyticus* and its biofilm formation.

Compared with physical sterilization methods (such as ultrahigh pressure, irradiation, and ultra sonification), chemical approaches using antibiotics, preservatives, or antibacterial agents can produce marked effects without energy consumption and are consequently low-carbon and more economical. Particularly, antimicrobial compounds of natural origins have attracted growing attention in recent years because of their safety, satisfactory efficacy, and multifunctional characteristics. Hexanal is a phytochemical and has been identified in different types of plants, including macroalgae, medicinal herbs, Myrtaceae plants, and fruits [11,12,13,14]. According to previous publications, hexanal showed antimicrobial activity against pathogenic fungi, such as *Aspergillus flavus*, *Penicillium expansum*, and *Botrytis cinerea*, and harmful bacteria, including *Escherichia coli*, *Pseudomonas fluorescens*, *Erwinia carotovora*, etc. [15,16,17,18]. Our previous study revealed the anti-*V. parahaemolyticus* activity of hexanal with an MIC value of 0.4 mg/mL [19]. However, changes in *V. parahaemolyticus* induced by hexanal at the cell level and the bacteriostatic application of hexanal in food systems have not been investigated. Also, the effect of hexanal on *V. parahaemolyticus* biofilm formation remains unclear.

In this study, the anti-*V. parahaemolyticus* mechanism of hexanal at the cellular level was explored by determinations of the time–kill curve and alterations in protein leakage, lactic dehydrogenase (LDH) release, intracellular ATP concentration, membrane integrity, cell morphology, and cell ultrastructure. In addition, the bacteriostatic efficacy of hexanal in tested seafood systems (Spanish mackerel meat and shrimp paste) was evaluated. Finally, the antibiofilm activity of hexanal against *V. parahaemolyticus* was examined via a crystal violet (CV) staining assay, SEM observation, quantification of extracellular protein and polysaccharide, determination of intracellular c-di-GMP level, and RT-qPCR analysis.

## 2. Materials and Methods

### 2.1. Reagents

Hexanal (GC: 99%, CAS: 66-25-1) was acquired from Macklin Biochemical Technology Co., Ltd. (Shanghai, China) and stored at 4 °C before use. Dimethyl sulfoxide (DMSO) was used to co-dissolve hexanal to the final concentration of 1% (*v*/*v*), which has been demonstrated to exert no inhibitory effect on *V. parahaemolyticus* growth. Other chemicals were of analytical grade.

### 2.2. Bacterial Strains and Culture Conditions

*V. parahaemolyticus* ATCC 17802 was purchased from the American Type Culture Collection (Manassas, VA, USA) and first activated on Luria–Bertani (LB) agar containing 3% NaCl. The resulting colonies were inoculated into LB broth containing 3% NaCl and incubated at 37 °C to the logarithmic phase. Cultures were centrifuged (4 °C, 5000× *g*, 5 min), washed twice with sterile saline, and resuspended to obtain an appropriate concentration for use.

### 2.3. Time–Kill Curves Determination

Briefly, the *V. parahaemolyticus* suspension (~10^6^ CFU/mL) was treated with hexanal (0, 1/2 MIC, 1MIC, and 2 MIC) and incubated at 37 °C for 24 h. At the time point of 0, 1, 2, 3, 6, 9, 12, and 24 h, samples were diluted with saline and spread onto 3% NaCl LB agar, followed by incubation and colony enumeration.

To examine the bactericidal effect of hexanal on *V. parahaemolyticus* in different medium systems, *V. parahaemolyticus* was inoculated into 3% NaCl LB broth, 3% NaCl aqueous solution, and 0.85% saline, respectively, to achieve a final bacterial concentration of 10^7^–10^8^ CFU/mL. The resultant suspensions were treated with hexanal (0, 2 MIC, and 4 MIC) at 37 °C for 3 h. At hourly intervals, the number of *V. parahaemolyticus* cells was enumerated as described above.

### 2.4. Lactic Dehydrogenase (LDH) Release Determination

According to the method of Yi et al. [20], the LDH release caused by hexanal was investigated. *V. parahaemolyticus* was suspended in sterile saline to a cell density of ~10 ^8^ CFU/mL and treated with hexanal at 0 (control), 2 MIC, and 4 MIC, followed by an incubation at 37 °C for 120 min. The samples were centrifuged (4 °C, 5000× *g*, 5 min), and the supernatant was collected for LDH determination using an LDH assay kit (Beyotime, Shanghai, China). The supernatant was mixed with LDH working solution in a 96-well plate according to the kit instructions. After 30 min-incubation at room temperature, absorbance at 490 nm was measured by a multimode reader (Spark, Tecan Austria GmbH, Groedig, Austria).

### 2.5. Protein Leakage Determination

The *V. parahaemolyticus* suspension (~10^8^ CFU/mL) was exposed to hexanal (0, 2 MIC, and 4 MIC) treatment for 180 min. At 30, 60, 90, 120, and 180 min, the samples were centrifuged at 10,000× *g* for 10 min, and the resulting supernatant was retained for use. Following the instruction manual of a BCA protein assay kit (Beyotime, Shanghai, China), the BCA working solution was prepared and then mixed with the supernatant. Subsequently, the samples were incubated at 37 °C for 30 min, and the absorbance values at 562 nm (A_562nm_) were read using a multimode reader (Spark, Tecan Austria GmbH, Groedig, Austria). The linear correlation between the A_562nm_ value and protein concentration was obtained, and the protein concentration was calculated using the standard curve method.

### 2.6. Intracellular ATP Concentration Determination

As previously described [21], the *V. parahaemolyticus* ATCC 17,802 suspension treated with hexanal at 0 (control), 2 MIC, and 4 MIC was incubated at 37 °C for 30 min. The samples were then centrifuged (4 °C, 5000× *g*, 5 min), and the obtained cells were exposed to lysis buffer for 10 min on ice with a discontinuous vortex. After another centrifugation (4 °C, 12,000× *g*, 5 min), the supernatant was collected for ATP concentration measurement following the instructions in the enhanced ATP assay kit (Beyotime, Shanghai). The luminescence of the samples was determined using a multimode microplate reader (Spark, Tecan Austria GmbH, Groedig, Austria).

### 2.7. Confocal Laser Scanning Microscope (CLSM) Observation

CLSM observation was performed to assess the effect of hexanal on *V. parahaemolyticus* membrane integrity. Briefly, *V. parahaemolyticus* cells treated with hexanal (0, 2 MIC, and 4 MIC) were incubated at 37 °C for 120 min, followed by staining with propidium iodide (PI) and SYTO 9. After being kept in the dark for 15 min, the samples were washed and suspended with sterile saline for use. Finally, a confocal laser scanning fluorescence microscope (Leica TCS SP8, Wetzlar, Germany) was used to observe the *V. parahaemolyticus* samples dropped onto glass slides.

### 2.8. Scanning Electron Microscope (SEM) and Transmission Electron Microscope (TEM) Observation

The *V. parahaemolyticus* cells were treated with hexanal (0, 2 MIC, and 4 MIC) for 120 min and rinsed with sterile saline before immersion in 2.5% glutaraldehyde at 4 °C. Subsequently, cells were fully washed and dehydrated in an ethanol solution (30%, 50%, 70%, 80%, 90%, and 100%). The *V. parahaemolyticus* samples were dried and gold sprayed before the final observation using a scanning electron microscope (Nano SEM-450, FEI, Hillsboro, USA). For TEM observation, the *V. parahaemolyticus* samples were fixed with 1% osmic acid for another 3 h before washing and dehydration. Then, the samples were permeated with alcohol/LR-white mixture, embedded in special capsules, dried at 55 °C for 48 h, and cut into ultrathin sections. After uranium–lead double staining, the samples were observed by a transmission electron microscope (TECNAI G2 SPIRIT, FEI, Hillsboro, USA).

### 2.9. Determianation of Antibacterial Efficacy in Food System

The antibacterial activity of hexanal against *V. parahaemolyticus* in Spanish mackerel meat and shrimp paste was evaluated using the method reported by Ning et al. [22], with some modifications. Briefly, the *V. parahaemolyticus* suspension and Spanish mackerel meat stuffing or shrimp paste were mixed in equal amounts and fully stirred to obtain homogeneous samples. Hexanal was added to Spanish mackerel meat samples to achieve final concentrations of 0 (control), 2 MIC, 4 MIC, 8 MIC, and 16 MIC, respectively. The resultant samples were incubated at 4 °C and 15 °C. At 0, 6, 12, 24, 36, 48, 60, and 72 h, the samples were diluted and spread onto TCBS agar for *V. parahaemolyticus* enumeration. The final concentrations of hexanal in shrimp paste samples were 0 (control), 2 MIC, 3MIC, 4 MIC, and 8 MIC, respectively. The samples were incubated at 4 °C and 15 °C for 0, 1, 2, 4, 6, and 9 h. At each sampling point, *V. parahaemolyticus* enumeration was conducted as described above.

### 2.10. Anti-Biofilm Activity Determinations

#### 2.10.1. Crystal Violet (CV) Staining Assay

The assay was performed according to the modified version of Kim et al. [23] to evaluate the effect of hexanal on *V. parahaemolyticus* biofilm formation. Hexanal was added into *V. parahaemolyticus* suspensions in 96-well microplates to obtain final concentrations of 0 (control), 1/8 MIC, or 1/4 MIC, and the resulting samples were incubated at 37 °C for 48 h. Subsequently, wells were rinsed three times, air-dried at 60 °C, and stained with CV solution (1%) for 30 min. After washing, wells were air-dried and dissolved with 33% glacial acetic acid. Finally, the absorbance at 570 nm (A_570_) was determined by a multimode reader, and the relative biofilm formation was expressed as A_treatment-570_/A_control-570_.

#### 2.10.2. SEM Observation

As previously reported [24], SEM observation was carried out to visualize *V. parahaemolyticus* biofilm formation in the absence or presence of hexanal. Briefly, *V. parahaemolyticus* suspensions treated with hexanal (0, 1/8 MIC, and 1/4 MIC) were added to 24-well plates containing a sterile glass slide (φ10 mm) in each well and cultured at 37 °C for 48 h. The biofilms that formed on glass slides were gently rinsed, air-dried, and suspended in 2.5% glutaraldehyde. Subsequent dehydration, drying, and observation were the same as described in Section 2.8.

#### 2.10.3. Quantification of Extracellular Protein and Polysaccharide

The quantification assay was performed following the method reported by Kim et al. [23] with some modifications. The *V. parahaemolyticus* suspensions were co-cultured with hexanal (0, 1/8 MIC, and 1/4 MIC) at 37 °C for 48 h. To extract extracellular polymeric substances, the samples were centrifuged (8000× *g*, 10 min), and the resulting cells were suspended in 1.5 M NaCl solution [25], followed by another centrifugation to harvest the supernatants. Finally, the contents of polysaccharide and protein were quantified using phenol–sulfuric acid method and the BCA protein assay kit, respectively.

#### 2.10.4. Determination of Intracellular c-di-GMP Level

The intracellular c-di-GMP concentration was measured as previously described [26]. The *V. parahaemolyticus* suspensions were co-cultured with hexanal (0, 1/8 MIC, and 1/4 MIC) at 37 °C for 48 h. After centrifugation and washing, the *V. parahaemolyticus* cells were suspended in ice-cold saline and incubated at 100 °C for 5 min. The samples were sonicated (100 w, on/2 s, off/2 s) in an ice-water bath for 6 min and centrifuged at 4 °C, 8000× *g* for 5 min. The supernatant was collected, and the cell sediments were sonicated another two times to fully extract the intracellular c-di-GMP. The total protein and c-di-GMP levels in the supernatant were determined using the BCA protein assay kit and the Enzyme-Linked Immunosorbent Assay kit (Beyotime, Beijing, China), respectively. Finally, the intracellular c-di-GMP concentration was expressed as pmol/mg protein.

#### 2.10.5. Isolation of RNA and RT-qPCR

The *V. parahaemolyticus* suspensions were co-cultured with hexanal (0, 1/8 MIC, and 1/4 MIC) at 37 °C for 48 h. Following the manual of the TIANGEN RNAprep Pure Cell/Bacteria Kit (Tiangen, Beijing), total RNA was extracted, and the concentration of RNA was measured and adjusted. An Evo M-MLV RT kit (AG, Hunan, China) was used to reverse transcribe RNA into cDNA. The primers used in this study are listed in Table 1. RT-qPCR was performed using a SYBR Green Premix Pro Taq HS qPCR kit (AG, Hunan, China) on a CFX96 PCR detection system. The expression levels of genes associated with biofilms were calculated by the 2^−ΔΔCT^ method and normalized to that of *puvA*.

### 2.11. Statistical Analyses

All experiments were performed in triplicate, and the data are presented as the mean ± standard deviation. The SPSS software (version 20.0, IBM-SPSS Inc., USA) was used to perform repeated measures analysis of variance (ANOVA), one-way ANOVA, and the Duncan test for significant difference (*p* < 0.05) analysis.

## 3. Results

### 3.1. Time-Kill Curves

**Figure 1:**
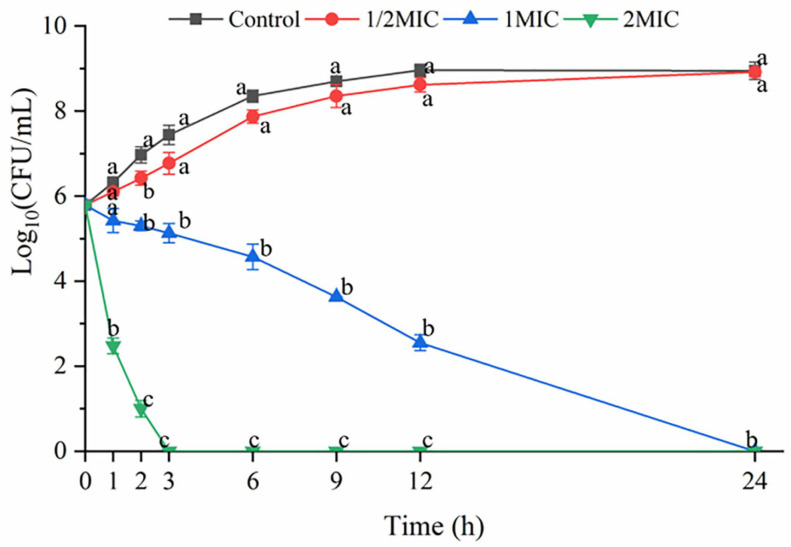
Time-kill curve of hexanal against *V. parahaemolyticus*. Different letters at the specific time points indicate significant differences compared with the control group (*p* < 0.05).

As shown in Figure 1, hexanal at 1/2 MIC did not inhibit bacterial growth, and the number of *V. parahaemolyticus* consistently increased during the monitored period. By contrast, hexanal at 1MIC and 2 MIC exerted distinct bactericidal effects against *V. parahaemolyticus*. The population of *V. parahaemolyticus* declined below the detection limit after treatment with hexanal (1MIC and 2 MIC) for 24 h and 3 h, respectively. The result confirmed that hexanal at 0.4 mg/mL was able to inhibit the growth of *V. parahaemolyticus*, which was consistent with the MIC value determined above.

**Figure 2:**
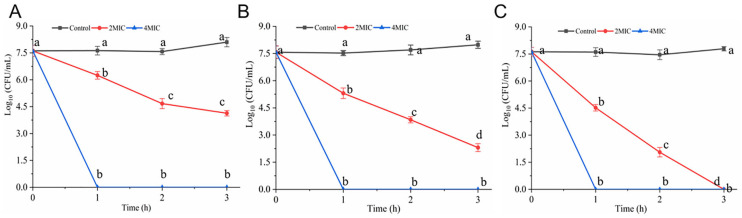
Inactivation effect of hexanal on *V. parahaemolyticus* in (**A**) 3% NaCl LB broth, (**B**) 3% NaCl solution, and (**C**) 0.85% saline. Different letters at four time points indicate significant differences compared with the initial number of *V. parahaemolyticus* (*p* < 0.05).

As presented in Figure 2A–C, hexanal showed different bactericidal efficacy in different suspension systems. The initial concentrations of *V. parahaemolyticus* in three systems (3% NaCl LB broth, 3% NaCl solution, and 0.85% saline) were 7.61, 7.57, and 7.62 log CFU/mL, which declined to 6.25, 5.31, 4.52 log CFU/mL and 4.67, 3.84, 2.06 log CFU/mL after exposure to hexanal (2 MIC) for 1 h and 2 h, respectively (*p* < 0.05). At 3 h, hexanal at 2 MIC reduced the cell numbers in 0.85% saline below the detection limit (Figure 2C). Overall, hexanal (2 MIC) showed the highest bactericidal efficacy against *V. parahaemolyticus* in 0.85% saline, followed by that in 3% NaCl aqueous solution and 3% NaCl LB broth. Furthermore, hexanal at 4 MIC killed all the *V. parahaemolyticus* cells in three systems in one hour.

### 3.2. Lactic Dehydrogenase (LDH) Release

**Figure 3:**
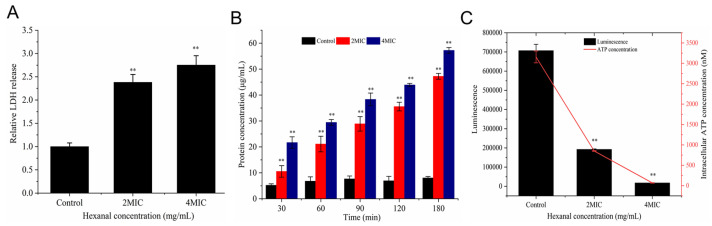
Effect of hexanal on (**A**) lactic dehydrogenase release, (**B**) protein leakage, and (**C**) intracellular ATP concentration of *V. parahaemolyticus*. **: *p* < 0.01, compared with the control group.

LDH is a stable enzyme in the cytoplasm and will leak out of cells when the membrane is destroyed [20]. As illustrated in Figure 3A, hexanal treatment significantly increased the release of LDH in *V. parahaemolyticus* (*p* < 0.01). LDH release in 2 MIC- and 4 MIC-treated samples was 2.38- and 2.75-fold that of the control. The increased LDH release indicated that hexanal may disrupt the cell membrane of *V. parahaemolyticus*.

### 3.3. Protein Leakage

The leakage of protein induced by hexanal is presented in Figure 3B. At 30 min, the concentrations of leaked protein in 2 MIC- and 4 MIC-treated samples were 10.56 and 21.67 μg/mL, respectively, and the levels continuously increased with the treatment time. At 90, 120, and 180 min, the leakage of protein caused by hexanal at 2 MIC was 28.89, 35.56, and 47.23 μg/mL, respectively. As hexanal increased to 4 MIC, higher levels of protein leakage (38.33, 43.89, and 57.22 μg/mL) were detected.

### 3.4. Intracellular ATP Concentration

The effect of hexanal on intracellular ATP concentration is shown in Figure 3C. Intracellular ATP concentration of the untreated group was 3157.94 nM, which reduced to 850.77 and 67.97 nM after *V. parahaemolyticus* was treated with hexanal at 2 MIC and 4 MIC, respectively (*p* < 0.05). The result suggested that hexanal treatment caused significant decreases in *V. parahaemolyticus* intracellular ATP concentration.

### 3.5. CLSM Observation

**Figure 4:**
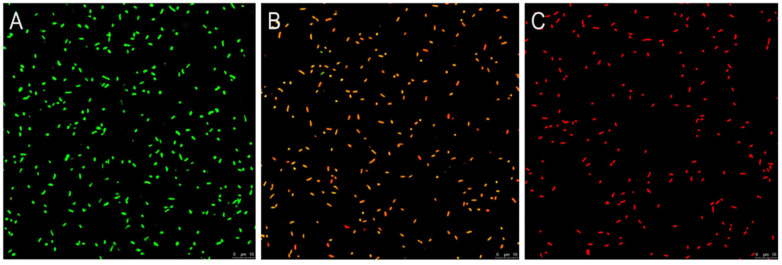
CLSM images of *V. parahaemolyticus* treated with hexanal at concentrations of 0 (**A**), 2 MIC (**B**), and 4 MIC (**C**). Scale bar: 10 μm. Green represents bacterial cells with intact cell membrane. Red represents cells with impaired cell membrane.

As depicted in Figure 4A, untreated *V. parahaemolyticus* samples showed complete and strong green fluorescence, suggesting intact cytomembranes and living cells, whereas cells exposed to hexanal (2 MIC) emitted orange or red fluorescence, which revealed that *V. parahaemolyticus* cells were increasingly losing membrane integrity and cell viability (Figure 4B). By comparison, *V. parahaemolyticus* in the 4 MIC-treated group exhibited strong red fluorescence, implying the damaged membrane and dead cells (Figure 4C).

### 3.6. SEM and TEM Observation

**Figure 5:**
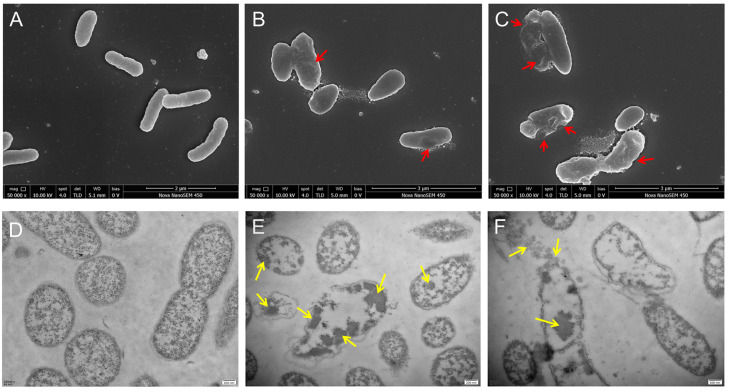
(**A**–**C**) SEM images and (**D**–**F**) TEM images of *V. parahaemolyticus* treated with hexanal at the concentrations of 0 (**A**,**D**), 2 MIC (**B**,**E**), and 4 MIC (**C**,**F**). For SEM images, the magnification was 5000×. For TEM images, the scale bar was 200 nm. Red arrows indicate cell shrinkages or cell depressions. Yellow arrows indicate cytoplasm aggregation or leakage.

*V. parahaemolyticus* cells without hexanal treatment were plump, three-dimensional, and exhibited short-rod morphology (Figure 5A). Also, the cell boundary was clear, and the cytoplasmic region was dense and well-distributed (Figure 5D), whereas cells treated with hexanal (2 MIC) became irregular, rough, and less full in shape (Figure 5B), and the cytoplasm was aggregated and uneven (Figure 5E). As the dosage of hexanal increased to 4 MIC, the *V. parahaemolyticus* cells lost normal morphology, and cell collapse, shrinkage, and depression were easily observed (Figure 5C). Moreover, the intracellular substance in some cells was obviously reduced and the leakage of cytoplasm seemed to be observed (Figure 5F).

### 3.7. Antibacterial Efficacy of Hexanal in Food System

**Figure 6:**
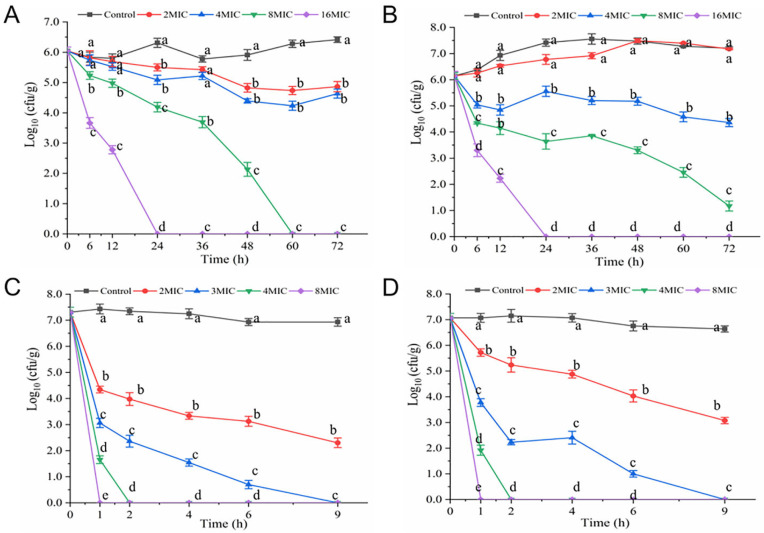
Antibacterial activity of hexanal against *V. parahaemolyticus* in Spanish mackerel meat at (**A**) 4 °C and (**B**) 15 °C. Antibacterial activity of hexanal against *V. parahaemolyticus* in shrimp paste at (**C**) 4 °C and (**D**) 15 °C. Different letters at the specific time points indicate significant differences compared with the control group (*p* < 0.05).

As presented in Figure 6A,B, the initial number of *V. parahaemolyticus* cells in Spanish mackerel meat was about 6.0 log CFU/g. At 4 °C, hexanal (2 MIC, 4 MIC, 8 MIC, and 16 MIC) resulted in an obvious decline in the number of *V. parahaemolyticus* (Figure 6A). To be specific, hexanal at 2 MIC and 4 MIC had similar efficacy and diminished *V. parahaemolyticus* cells to 4.87 and 4.63 log CFU/g after 72 h treatment. At 60 h and 24 h, bacterial counts in hexanal- (8 MIC and 16 MIC) treated samples declined below the detection limit (Figure 6A). At 15 °C, hexanal at 2 MIC was unable to inhibit bacterial growth in samples. Hexanal at 4 MIC, 8 MIC, and 16 MIC showed a similar inactivation effect on *V. parahaemolyticus* (Figure 6B).

As illustrated in Figure 6C,D, the number of *V. parahaemolyticus* in shrimp paste samples rapidly decreased after exposure to hexanal. At 9 h, cell counts in 2 MIC hexanal-treated shrimp paste (at 4 °C and 15 °C) were 2.30 and 3.07 log CFU/g, respectively. Hexanal at 3MIC, 4 MIC, and 8 MIC reduced the cell number below the detection limit after 9 h, 2 h, and 1 h of treatment, respectively. Overall, hexanal exhibited a good inactivation effect on *V. parahaemolyticus* in shrimp paste.

### 3.8. Anti-Biofilm Effect of Hexanal on V. parahaemolyticus

#### 3.8.1. Crystal Violet (CV) Staining

**Figure 7:**
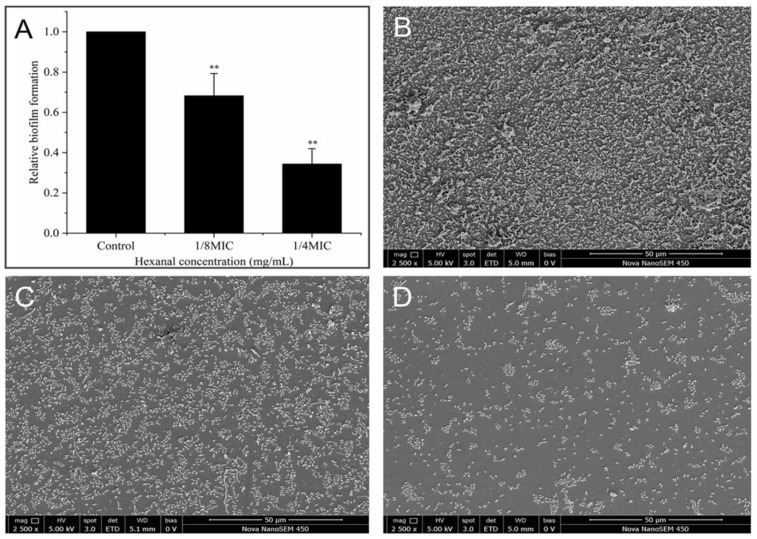
(**A**) Effect of hexanal on biofilm formation of *V. parahaemolyticus*. **: *p* < 0.01, compared with the control group. (**B**–**D**) SEM images of *V. parahaemolyticus* biofilm formation in the presence of hexanal at 0 (**B**), 1/8 MIC (**C**), and 1/4 MIC (**D**).

The results of the CV staining assay are presented in Figure 7A. It is evident that hexanal was effective in inhibiting biofilm formation by *V. parahaemolyticus*. Compared with the control, *V. parahaemolyticus* biofilm formation in the presence of hexanal (1/8 MIC and 1/4 MIC) decreased by 31.8% and 65.4%, respectively (*p* < 0.05), indicating the antibiofilm potential against *V. parahaemolyticus.*

#### 3.8.2. SEM Observation for Biofilm Formation

To visualize the effect of hexanal on *V. parahaemolyticus* biofilm formation, SEM observation was conducted, and the images of biofilms are shown in Figure 7B–D. Without hexanal treatment, *V. parahaemolyticus* established a dense and uniform cell layer with many bacterial clusters inside (Figure 7B). In contrast, in the presence of 1/8 MIC-hexanal, *V. parahaemolyticus* formed a thinner, discontinuous, and less well-distributed biofilm layer (Figure 7C). As the dosage of hexanal increased to 1/4 MIC, the number of cells in the layer evidently decreased, and the cell aggregations also became fewer (Figure 7D). These SEM images confirmed that hexanal was effective in inhibiting *V. parahaemolyticus* biofilm formation.

#### 3.8.3. Extracellular Protein and Polysaccharide

**Figure 8:**
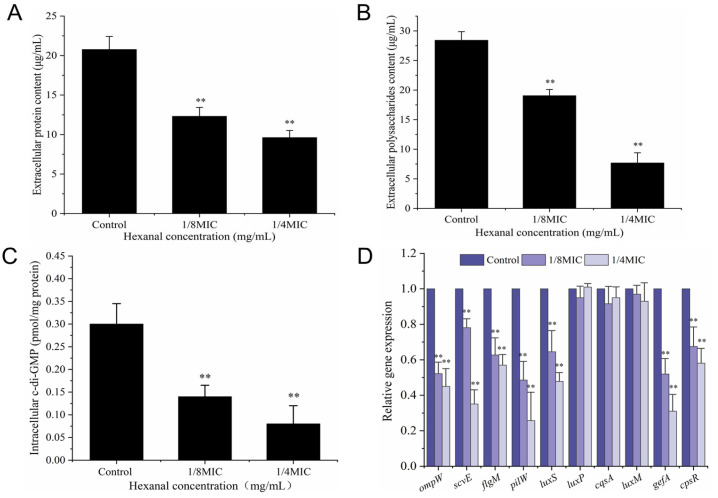
(**A**) Extracellular protein production and (**B**) extracellular polysaccharide production in *V. parahaemolyticus* treated with hexanal at 0, 1/8 MIC, and 1/4 MIC. (**C**) Intracellular c-di-GMP level in *V. parahaemolyticus* treated with hexanal at 0, 1/8 MIC, and 1/4 MIC. (**D**) Relative expression levels of biofilm-related genes in *V. parahaemolyticus* treated with hexanal at 0, 1/8 MIC, and 1/4 MIC. **: *p* < 0.01, compared with the control group.

As presented in Figure 8A,B, the concentrations of extracellular protein and polysaccharide in untreated *V. parahaemolyticus* were 20.78 and 28.42 μg/mL, respectively, which dropped to 12.31 and 21.65 μg/mL in the presence of hexanal at 1/8 MIC (*p* < 0.05). Moreover, lower protein and polysaccharide levels (9.62 and 7.68 μg/mL) were determined in 1/4 MIC hexanal-treated samples. The results revealed that hexanal significantly reduced the contents of protein and polysaccharide (the essential constituents of biofilms), exerting an inhibitory effect on *V. parahaemolyticus* biofilm formation.

#### 3.8.4. Intracellular c-di-GMP Level

The secondary messenger c-di-GMP ubiquitous in bacteria is a core regulatory factor for biofilm formation and also plays important roles in other bacterial cellular pathways, including virulence, motility, and drug resistance [26]. As shown in Figure 8C, the intracellular c-di-GMP concentration in untreated *V. parahaemolyticus* was 0.30 pmol/mg protein, which significantly decreased to 0.14 and 0.08 pmol/mg protein in the presence of hexanal at 1/8 MIC and 1/4 MIC, respectively (*p* < 0.05). The results indicated that hexanal was effective in reducing the production of intracellular c-di-GMP, which finally led to the inhibition of biofilm formation.

#### 3.8.5. RT-qPCR Analysis

An RT-qPCR assay was performed to evaluate the effect of hexanal on the transcription levels of biofilm-related genes. As depicted in Figure 8D, the expression of *ompW*, *scvE*, *flgM*, *pilW*, *luxS*, *gefA*, *cpsR* in 1/8 MIC-hexanal treated *V. parahaemolyticus* was significantly downregulated, with the reductions of 47.80%, 21.95%, 37.58%, 51.47%, 35.53%, 48.32%, and 32.50%, respectively (*p* < 0.05). In *V. parahaemolyticus* samples exposed to hexanal at 1/4 MIC, higher downregulations of the above genes were achieved. Furthermore, the transcription levels of *luxP*, *luxM*, and *cqsA* remained unchanged upon hexanal treatment, indicating the three genes were not involved in the biofilm formation inhibition mediated by hexanal.

## 4. Discussion

In recent years, compounds of natural origin with antimicrobial activity have received growing attention from researchers due to their high antimicrobial efficiency, safety, and other advantages. Many of the compounds displayed great potential to control pathogenic microorganisms. Hexanal is a plant-derived aldehyde with a “green” note and is also the main flavor component of seafood [18,27]. One of our recent studies found that hexanal exerted anti-*V. parahaemolyticus* activity with an MIC of 0.4 mg/mL [19]. In order to better apply hexanal for *V. parahaemolyticus* control, in this study, we performed time-killing assays to determine the needed dosage and time for hexanal to inactivate *V. parahaemolyticus* cells. It was evident from Figure 1 that hexanal at 2 MIC had the potential for *V. parahaemolyticus* inactivation in 3 h. As shown in Figure 2, hexanal at 2 MIC and 4 MIC was effective in inactivating *V. parahaemolyticus* in three suspension systems, and the best bactericidal efficacy was observed in 0.85% saline, followed by that in 3% NaCl LB broth and 3% NaCl solution. The difference in bactericidal efficacy may be associated with the resistance of *V. parahaemolyticus* in different suspending media.

In order to reveal the anti-*V. parahaemolyticus* mechanism of hexanal at the cellular level, we investigated the alternations in LDH release, protein leakage, membrane integrity, cell morphology, and cell ultrastructure caused by hexanal, aiming to provide a theoretical foundation for the real application of hexanal to kill *V. parahaemolyticus* in food field. LDH release and protein leakage are common indicators used to reflect whether the cell membrane is injured. When the cytoplasmic membrane is disrupted or its permeability increases, the cytoplasmic content, including LDH and some proteins, is released into the extracellular medium [20,28]. After treatment with hexanal, LDH release and protein leakage of *V. parahaemolyticus* were significantly increased (Figure 3A,B), revealing that the cell membrane of *V. parahaemolyticus* might be damaged. Similar to our study, Yi et al. [20] treated *Listeria monocytogenes* and *Cronobacter sakazakii* with a bacteriocin and found the release of LDH was enhanced. Wang et al. [28] explored the antibacterial mechanism of lactic acid and demonstrated that lactic acid caused increased leakage of protein in *Salmonella Enteritidis*, *E. coli,* and *L. monocytogenes*. To further confirm the effect of hexanal on the *V. parahaemolyticus* membrane, a fluorescence-based membrane integrity test was conducted under CLSM using a LIVE/DEAD^®^ BacLight^TM^ Kit (Thermo, Waltham, MA, USA). PI and SYTO 9 in the kit are nucleic acid-binding dyes and have gained extensive application in the characterization of membrane damage. In hexanal-treated samples, green fluorescence almost disappeared, and the intensity of red fluorescence was rapidly enhanced with the increase in hexanal concentration (Figure 4), implying that hexanal damaged the integrity of the *V. parahaemolyticus* cytoplasmic membrane in a dose-dependent manner and lead to the loss of cell viability. The result was similar to that of thymol against *Enterobacter sakazakii* and that of protocatechuic acid against *Yersinia enterocolitica* [29,30]. SEM and TEM are powerful tools for visually observing the effect of hexanal on *V. parahaemolyticus* morphology and ultrastructure. As illustrated in Figure 5, hexanal at 2 MIC and 4 MIC caused irregular, shrunken, and ruptured *V. parahaemolyticus* cells with aggregated cytoplasm and leaked cell components. The results were in accordance with that of CLSM observation and further confirmed the destructive effect of hexanal on *V. parahaemolyticus* cells.

To evaluate the antibacterial efficiency of hexanal against *V. parahaemolyticus* in the food system, we inoculated *V. parahaemolyticus* into Spanish mackerel meat and shrimp paste samples, treated them with hexanal, and monitored the resultant *V. parahaemolyticus* growth. As shown in Figure 6, hexanal was effective in reducing the amount of *V. parahaemolyticus* in Spanish mackerel meat and shrimp paste samples. Moreover, the inactivation efficacy of hexanal against *V. parahaemolyticus* in shrimp paste was higher than that in Spanish mackerel meat. The finding indicated that *V. parahaemolyticus* cells in shrimp paste appeared to be more sensitive to hexanal than that in Spanish mackerel meat. Taken together, it can be concluded that the anti-*V. parahaemolyticus* efficacy of hexanal in different food systems was discrepant. Similar to our study, the antibacterial activity of ε-poly-lysine, phenyllactic acid, and blueberry extract against *V. parahaemolyticus* in food systems at 4 °C or 25 °C was investigated [22,31,32]. The current preliminary study assessed the antibacterial effects of hexanal against *V. parahaemolyticus* in two food systems and showed that hexanal has the potential to be used as an antibacterial agent. However, further in-depth explorations are required before the final practical application.

Biofilm formation enhances the difficulty of *V. parahaemolyticus* control and has been demonstrated to be the major cause of seafood cross-contamination. In this study, the antibiofilm activity of hexanal against *V. parahaemolyticus* was evaluated by determining the alterations in biofilm biomass, biofilm morphology, extracellular protein and polysaccharide production, intracellular c-di-GMP level, and biofilm-associated gene expression. CV staining assay suggested that hexanal at 1/8 MIC and 1/4 MIC significantly reduced biofilm formation of *V. parahaemolyticus* (Figure 7A), which was confirmed by SEM images of *V. parahaemolyticus* biofilms formed in the absence or presence of hexanal (Figure 7B–D). As previously reported, many plant-derived compounds exert antibiofilm activity against *V. parahaemolyticus*. For example, protocatechuic aldehyde, eugenol, and Laurel essential oil were proven to inhibit the biofilm formation of *V. parahaemolyticus*, with a visually reduced biofilm biomass [33,34,35]. Extracellular proteins and polysaccharides are the main components of bacterial biofilms and play important roles in maintaining biofilm structure [36]. In this study, hexanal at 1/8 MIC and 1/4 MIC significantly reduced the contents of extracellular proteins and polysaccharides in *V. parahaemolyticus* (Figure 8A,B). The results revealed that the inhibition of biofilm formation may be attributed to the potential of hexanal to restrain the production of extracellular proteins and polysaccharides. Similarly, Zhu et al. [37] demonstrated that sodium butyrate at sub-inhibitory concentrations reduced the secretion of extracellular proteins and polysaccharides, leading to a loosely structured *V. parahaemolyticus* biofilm with fewer constituents.

C-di-GMP is a second messenger that extensively exists in bacteria and is involved in the regulation of various cellular behaviors, including biofilm formation [38]. It has been reported that high levels of intracellular c-di-GMP contributed to bacterial surface attachment and biofilm formation while inhibiting cell motility [39]. In this study, the intracellular c-di-GMP level of *V. parahaemolyticus* decreased in the presence of hexanal at 1/8 MIC and 1/4 MIC (Figure 8C), suggesting that hexanal affected the metabolism of c-di-GMP and finally led to biofilm formation inhibition against *V. parahaemolyticus*. Biofilm formation is a complex process that involves multiple factors and genes, such as quorum sensing (QS) signaling molecules and their regulatory genes [40]. In order to better clarify the antibiofilm mechanism of hexanal, we investigated alternations in the expression levels of *V. parahaemolyticus* genes involved in cell motility (*flgM*, *pilW*), extracellular matrix production (*ompW*, *scvE*, *cpsR*), and quorum sensing (*luxS*, *luxP*, *luxM*, *cqsA*) [41,42,43]. As presented in Figure 8D, hexanal at 1/8 MIC and 1/4 MIC significantly reduced the expression levels of genes (*ompW*, *scvE*, *flgM*, *pilW*, *luxS*, *gefA*, *cpsR*) associated with biofilm development, suggesting the antibiofilm potential of hexanal against *V. parahaemolyticus.* It is well known that *luxS*, *luxM*, and *cqsA* are the corresponding encoding genes of three QS signal synthases (LuxS, LuxM, and CqsA) in *V. parahaemolyticus*, which regulates the synthesis of three QS signaling molecules AI-2, HAI-1, and CAI-1, respectively [42]. In this study, hexanal (1/8 MIC and 1/4 MIC) exerted no effect on the transcription levels of *luxM* and *cqsA* (Figure 8D), which implies that QS systems mediated by LuxM and CqsA may not be involved in the repression of *V. parahaemolyticus* biofilm formation caused by hexanal. Collectively, the RT-qPCR analysis revealed that hexanal inhibited biofilm formation of *V. parahaemolyticus* by affecting cell motility, extracellular matrix production, and *luxS-*mediated quorum sensing. However, the specific pathways and targets remain unclear. Therefore, further studies are required to fully elucidate the antibiofilm mechanism of hexanal against *V. parahaemolyticus.*

## 5. Conclusions

To summarize, hexanal at 2 MIC and 4 MIC was effective in inhibiting *V. parahaemolyticus* growth. Hexanal exerted an antibacterial effect on *V. parahaemolyticus* by damaging the cell membrane, further causing increased protein leakage and LDH release, impaired membrane integrity, as well as abnormal morphology and cell ultra-structure. Also, hexanal was able to inactivate *V. parahaemolyticus* in Spanish mackerel meat and shrimp paste. Furthermore, hexanal exhibited antibiofilm activity against *V. parahaemolyticus* at 1/8 MIC and 1/4 MIC, as evidenced by the reduced biofilm formation, the declined production of extracellular polysaccharide and protein, the decreased intracellular c-di-GMP levels, and the downregulation of biofilm-associated genes. Our study suggested that hexanal has the potential as an alternative strategy to combat *V. parahaemolyticus* and its biofilm formation, thus reducing the contamination and foodborne illness caused by *V. parahaemolyticus*. Further investigations concerning the antibiofilm mechanism of hexanal against *V. parahaemolyticus* are still required.

## Figures and Tables

**Table 1:** Primers used for RT-qPCR in this study.

Genes	Primer	Sequences (5′–3′)
*puvA*	Forward	CAAACTCACTCAGACTC
	Reverse	CGAACCGATTCAACAC
*ompW*	Forward	TCGTGTCACCAAGTGTTTTCG
	Reverse	CGTGGCTGAATGGTGTTGC
*scvE*	Forward	GACAGGTCGTGATGCCATTC
	Reverse	GGCGATGATGACCGAAGTG
*flgM*	Forward	TTGATCGTGCCCAAGCAGAA
	Reverse	TCTAGGCTCAATTCGCCGC
*pilW*	Forward	AGCTCCTATCGTGAAAGCCG
	Reverse	AAGCTGTGCGCGGTAGTATT
*luxS*	Forward	GCAGGGTTTGACTCCACACT
	Reverse	TGATGGCTGCTGCAATGAGT
*luxP*	Forward	GTTCTGCTGAGCTAGACGCTATC
	Reverse	AGTACACCGTTGGTACAGGTTTG
*cqsA*	Forward	ACTTCCACACTCAAGAGCAATA
	Reverse	GTTCAAGCGAGCCAAAGAAC
*luxM*	Forward	TGCCCTTGTTGTCACTTTCT
	Reverse	CGTTGGTTCCAGTCTTGGATTA
*gefA*	Forward	GCTTTACAACAACTACGTGG
	Reverse	GGTATCTGACAAAGTATCAC
*cpsR*	Forward	TGTCTAGCAACCGCACTAACC
	Reverse	GCTCTTACAACTCGGCTTCAC

## Data Availability

The original contributions presented in the study are included in the article. Further inquiries can be directed to the corresponding author.
